# Unraveling Light-Activated Insulin Action in Regulating Blood Glucose: New Photoactivatable Insight as a Novel Modality in Diabetes Management

**DOI:** 10.3390/molecules29061294

**Published:** 2024-03-14

**Authors:** Fahrul Nurkolis, Rudy Kurniawan, Elvan Wiyarta, Rony Abdi Syahputra, Reggie Surya, Nurpudji Astuti Taslim, Trina Ekawati Tallei, Raymond Rubianto Tjandrawinata, Eli Y. Adashi, Bonglee Kim

**Affiliations:** 1 Biological Sciences, Faculty of Sciences and Technology, State Islamic University of Sunan Kalijaga (UIN Sunan Kalijaga), Yogyakarta 55281, Indonesia; fahrul.nurkolis.mail@gmail.com; 2 Graduated School of Medicine, Faculty of Medicine, Hasanuddin University, Makassar 90245, Indonesia; 3 Diabetes Connection Care, Eka Hospital Bumi Serpong Damai, Tangerang 15321, Indonesia; 4 Department of Neurology, Faculty of Medicine, Universitas Indonesia-Dr. Cipto Mangunkusumo National Hospital, Jakarta 10430, Indonesia; 5 Department of Pharmacology, Faculty of Pharmacy, Universitas Sumatera Utara, Medan 20155, Indonesia; 6 Department of Food Technology, Faculty of Engineering, Bina Nusantara University, Jakarta 11480, Indonesia; 7 Division of Clinical Nutrition, Department of Nutrition, Faculty of Medicine, Hasanuddin University, Makassar 90245, Indonesia; 8 Department of Biology, Faculty of Mathematics and Natural Sciences, Universitas Sam Ratulangi, Manado 95115, Indonesia; 9 Department of Biotechnology, Faculty of Biotechnology, Atma Jaya Catholic University of Indonesia, Jakarta 12930, Indonesia; 10 Warren Alpert Medical School, Brown University, Providence, RI 02903, USA; 11 Department of Pathology, College of Korean Medicine, Kyung Hee University, Kyungheedae-Ro 26, Dongdaemun-gu, Seoul 05254, Republic of Korea

**Keywords:** blood glucose, photoactivatable drugs, insulin, diabetes, hyperglycemia

## Abstract

Diabetes, particularly type 2 diabetes (T2D), is the main component of metabolic syndrome. It is highly prevalent and has drastically increased with sedentary lifestyles, notably behaviors linked to ease of access and minimal physical activity. Central to this condition is insulin, which plays a pivotal role in regulating glucose levels in the body by aiding glucose uptake and storage in cells, and what happens to diabetes? In diabetes, there is a disruption and malfunction in insulin regulation. Despite numerous efforts, effectively addressing diabetes remains a challenge. This article explores the potential of photoactivatable drugs in diabetes treatment, with a focus on light-activated insulin. We discuss its advantages and significant implications. This article is expected to enrich the existing literature substantially, offering a comprehensive analysis of potential strategies for improving diabetes management. With its minimal physical intrusion, light-activated insulin promises to improve patient comfort and treatment adherence. It offers precise regulation and localized impact, potentially mitigating the risks associated with conventional diabetes treatments. Additionally, light-activated insulin is capable of explicitly targeting RNA and epigenetic factors. This innovative approach may pave the way for more personalized and effective diabetes treatments, addressing not only the symptoms but also the underlying biological causes of the disease. The advancement of light-activated insulin could revolutionize diabetes management. This study represents a pioneering introduction to this novel modality for diabetes management.

## 1. Introduction

In the era of modernization and digitalization, we have improved life standards due to socio-economic growth, but sedentary lifestyles and unhealthy diets have become more common. This has caused a surge in obesity and the diseases that come with it, such as type 2 diabetes (T2D) [1,2,3,4]. In T2D, insulin is used to control the blood glucose combined with other oral hypoglycemic medications when other drugs are unresponsive [5,6,7].

**Figure 1:**
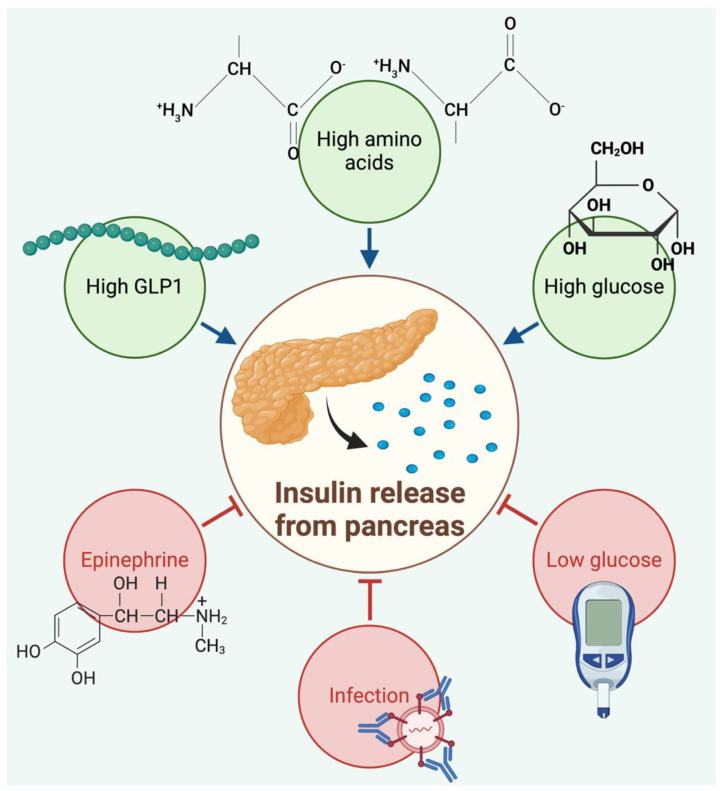
Regulation of insulin release. High levels of glucose, amino acids, and GLP1 result in the release of insulin, and this release is inhibited by conditions of high levels of epinephrine, low blood sugar levels, and the occurrence of infections. Created using Biorender Premium, licensed by Fahrul Nurkolis.

Insulin plays a central role in regulating glucose levels in the body by facilitating the uptake and storage of glucose in cells [8,9,10,11]. After glucose ingestion, insulin secretion is stimulated, leading to a series of metabolic responses that return blood glucose levels to normal (Figure 1). Insulin inhibits glucagon secretion and suppresses endogenous glucose production. Additionally, insulin influences adipose tissue metabolism by suppressing lipolysis and promoting glucose uptake [12,13,14]. Insulin also contributes to maintaining skeletal muscle mass by suppressing intramuscular protein breakdown rates. It stimulates vasodilation in arterial smooth muscle, enhancing blood flow, and the delivery of insulin and glucose to muscle cells [15,16,17]. Insulin is a relatively small protein with two peptide chains linked by disulfide bonds [18,19].

In T2D, insulin resistance occurs when cells, particularly the insulin receptors in the muscle, fat, and liver cells, do not respond adequately to insulin [20,21]. This interaction is caused by mutations, resulting in an altered insulin structure and causing its altered action, hyperinsulinemia, and the development of adult-onset diabetes with autosomal dominant inheritance [20,21]. The pancreas produces more insulin to counteract insulin resistance. Initially, this compensatory mechanism helps maintain normal blood glucose levels. However, prolonged insulin resistance and increased demand for insulin can lead to pancreatic beta-cell dysfunction and reduced insulin production [22]. This requires insulin therapy to manage the insufficient insulin to control blood glucose. Insulin therapy involves basal and rapid-acting insulin (RAI) therapy [23,24,25]. Currently, innovation is aimed at more personalized and convenient insulin therapies, catering to the diverse needs of individuals managing diabetes.

Adherence to insulin therapy is crucial to managing diabetes effectively [26,27]. For individuals with diabetes, particularly those with type 1 diabetes or advanced type 2 diabetes, insulin therapy plays a central role in regulating blood glucose levels. Adherence involves consistently following the prescribed insulin regimen, including the correct dosage, timing, and mode of administration. Proper adherence helps maintain stable blood sugar levels, preventing complications associated with high or low glucose levels. Factors influencing adherence include the complexity of the insulin regimen, fear of injections, and lifestyle considerations [28,29,30,31]. Hence, non-invasive insulin therapy increases adherence to therapy [32,33,34]. Several methods have been proposed, including oral and transdermal insulin administration. However, this is challenging as insulin has a significant molecular weight and can be broken down in the gastrointestinal system [35,36]. In addition, insulin administration is variable depending on the dietary habits of the patient. One recent medical advancement that has considerable potential is photoactivatable drugs. Photoactivatable drugs are linked to photoactive compounds that enable the modification or adjustment of the drug’s properties by using light or the controlled release of active components via a photorelease process [37,38,39,40,41,42,43,44]. Photoactivatable drugs are a promising treatment trend because they have fewer side effects and can hit RNA and epigenetic targets [45]. However, photoactivatable drugs, as a classical approach, are typically used for the development of new anti-cancer agents, and they have not yet been applied to other diseases, especially diabetes.

Despite advances in medical science and a broad spectrum of management strategies, current diabetes management methods have not curbed its increasing prevalence effectively. There is a pressing need to re-evaluate existing approaches, foster interdisciplinary collaboration, and invest in innovative research to develop more targeted interventions to mitigate the increase in cases of diabetes and its associated complications effectively. Therefore, this article aims to reveal the potential of photoactivatable drugs in treating diabetes by targeting light-activated insulin. Several advantages and potentially significant implications are also discussed. This research introduces a novel modality to diabetes management, and this contribution is anticipated to enhance the existing body of literature significantly by thoroughly examining potential strategies for advancing diabetes management.

## 2. Search Strategy and Selection Criteria

References for this review were identified via searches of PubMed for articles published until January 2024, using the search scope of “Articles published in English”. Relevant references cited in those articles were reviewed. The terms were as follows: ((diabetes) OR (insulin resistance)) OR ((diabetes mellitus) OR (type 2 diabetes); (insulin) AND ((photoactivated) OR (analogs) OR (synthetic) OR (isomerization) OR (isomer)); ((insulin) OR (insulin therapy) OR ((anti-diabetic drugs) OR (anti-diabetic medication))) AND (adherence). Relevant articles were identified by searching the authors’ files in Google Scholar, Scopus, ScienceDirect, and the Springer Online Archives Collection.

## 3. Insulin and Insulin Derivatives: What Happens to Them?

Insulin is a protein secreted by the beta cells of the pancreas as a response to glucose ingestion to return the plasma glucose concentration to normal within 2 h [15,19,35]. Insulin is a relatively small protein with two peptide chains linked by disulfide bonds. The primary structure of insulin involves a chain of 51 amino acids [46,47]. These amino acids are organized into an A-chain (21) and a B-chain (30), which are connected by two disulfide bonds between cysteine residues. The disulfide linkages contribute to the stability and three-dimensional structure of the insulin molecule [48,49].

**Figure 2:**
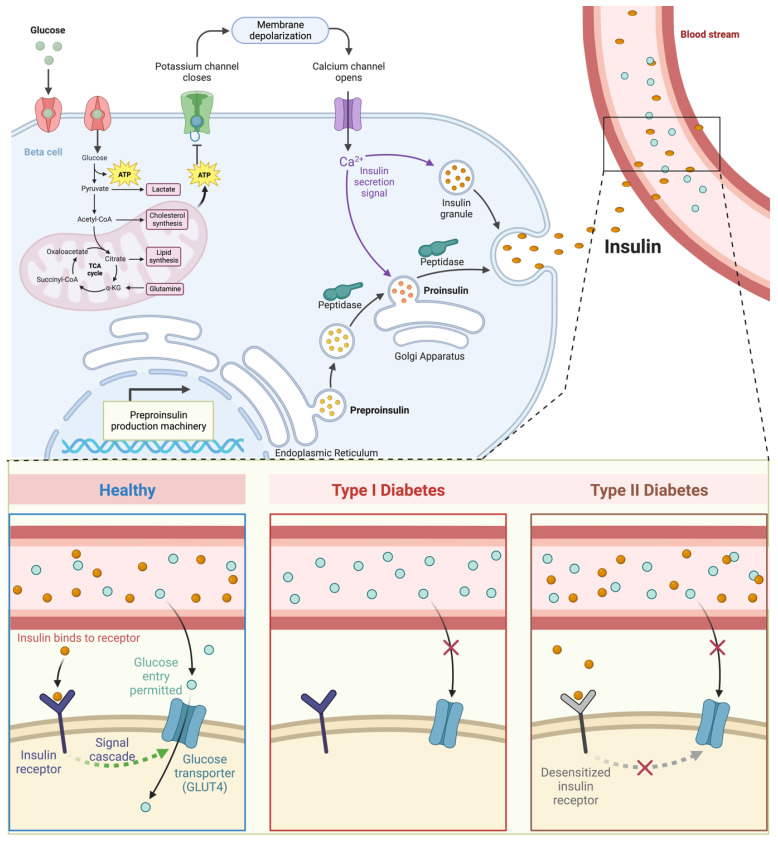
Mechanism of action, production, and glucose regulation by insulin. Insulin is responsible for helping glucose enter skeletal muscle cells to be metabolized into glycogen or synthesized into energy in the form of ATP, but in T2D and T1D this does not work. Created using Biorender Premium, licensed by Fahrul Nurkolis.

Insulin’s anabolic effects are exemplified by its role in regulating nutrient storage within skeletal muscle, the body’s primary reservoir of carbohydrates and protein [16,17]. This process involves insulin-mediated glucose uptake and amino acid transport into myocytes, orchestrated by the recruitment of glucose transporter 4 (GLUT4) to the plasma membrane (Figure 2). Insulin’s signaling cascade triggers the translocation of GLUT4 vesicles, facilitating glucose entry into muscle cells [50]. At the myocellular membrane, insulin engages with its receptor’s extracellular domain (α subunits), setting off a series of events [20,51]. This includes transphosphorylation of the intracellular β subunits and subsequent tyrosine phosphorylation of insulin receptor substrate (IRS) proteins [52,53]. The phosphorylated IRS-1 then binds to and activates phosphoinositide 3-kinase (PI3K), leading to the production of [3,4,5]-triphosphate (PIP3) [54,55]. This, in turn, triggers the downstream phosphorylation and activation of Akt (protein kinase B), a pivotal metabolic transducer [56,57]. In skeletal muscle, Akt phosphorylation is instrumental in facilitating the movement of intracellular GLUT4 vesicles [58,59]. This movement involves the translocation of GLUT4 from its intracellular location to the plasma membrane, allowing glucose to enter the muscle cells. Insulin also plays a crucial role in maintaining a glucose concentration gradient, supporting glucose phosphorylation to glucose-6-phosphate (G6P) via hexokinase II [15,60]. This phosphorylation, regulated by insulin, is essential for sustained facilitative glucose transport into muscle cells.

Furthermore, insulin regulates glucose metabolism pathways in skeletal muscle, influencing glucose oxidation, glycogen synthesis, and protein metabolism [15,61]. However, controversies persist regarding the absolute dependence of insulin-mediated GLUT4 translocation on critical components of the insulin-signaling pathway in muscle. Insulin-resistant skeletal muscle in type 2 diabetes is characterized by impaired non-oxidative glucose storage [62]. Additionally, insulin’s influence extends to regulating muscle protein metabolism and stimulating sodium–potassium ATPase activity [63].

Insulin’s crystal structure was first discovered to have zinc insulin hexamers [47,64,65,66,67]. This representation of a protein homo-oligomer was groundbreaking, offering a structural foundation for delving into the biosynthesis and storage of insulin within pancreatic β cells. Despite its small size (51 amino acids per monomer), the zinc hexamer showcases essential features standard to globular proteins, such as well-defined secondary structure elements, tertiary organization with a hydrophobic core, specific interfaces for self-assembly, and the capability for long-range conformational change, exemplified by the TR transition. The latter foreshadowed recent revelations about the structural mechanisms amplifying and transmitting conformational changes in insulin upon binding to the insulin receptor (IR) to affect transmembrane signaling [47,64,65,66,67].

**Figure 3:**
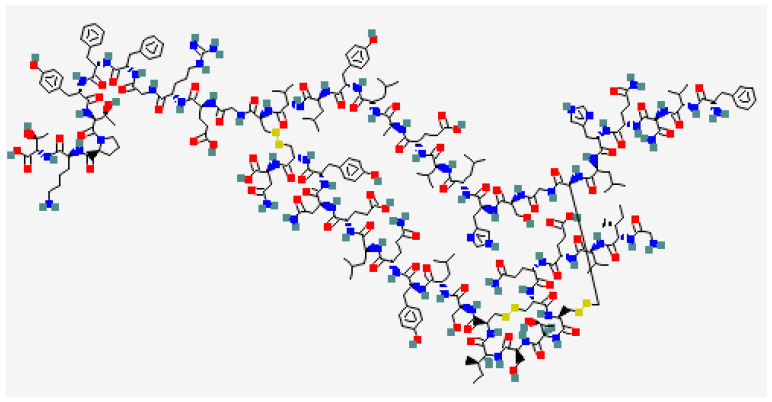
Molecular structure of insulin (PubChem CID: 70678557; https://pubchem.ncbi.nlm.nih.gov, accessed on 28 January 2024).

Insulin’s crystal structure laid the groundwork for optimizing its molecular properties for clinical applications (Figure 3). The refinement of the insulin molecule for the modulation of its pharmacokinetic (PK) and pharmacodynamic (PD) properties, seen in “first-generation” insulin analogs, was a pioneering achievement in rational protein design [68]. The current roster of products falls into two classes: rapid-acting formulations designed for bolus injection before meals, or for use in insulin pumps and long-acting formulations intended for once-a-day injection [68,69]. This array of products aims to replicate the homeostatic insulin secretion pattern by pancreatic β cells. The fundamental concept initially realized in the 1930s via examinations of microcrystalline suspensions proposed that the physical chemistry of insulin, including its self-association equilibria, directly influences the stability of a subcutaneous (SC) depot and its absorption rate [70,71,72,73]. Below, we discuss the first-generation prandial and basal analogs, with an emphasis on structure–function relationships.

The development of fast-acting insulin analogs was based on the idea that facilitating quicker disassembly within a subcutaneous (SC) depot could enhance the absorption of zinc-free insulin monomers and dimers into capillaries [74,75]. Various substitutions of amino acids at subunit interfaces have been investigated to weaken either dimerization or hexamer assembly. Candidate analogs were chosen based on their compatibility with high-affinity binding to the IR, maintenance of native in vivo activity, and suitability for stable pharmaceutical formulation. Notably, three currently employed fast-acting analogs—insulin lispro, insulin aspart, and insulin glulisine—have proven safe and effective in multi-injection regimens and continuous SC infusion, meeting established criteria for chemical and physical stability [76,77]. However, their vulnerability to degradation above room temperature necessitates ongoing efforts to fine-tune formulations for expedited SC absorption.

Insights from crystallographic studies of insulin lispro and aspart have unveiled mechanisms contributing to their rapid onset of action [70,78,79,80]. Hexamer assembly, which is critical for maintaining formulation stability, involves the specific binding of phenolic ligands, inducing an allosteric transition from T to R conformation in zinc insulin hexamers. The subsequent release of these ligands allows unliganded variant hexamers to disassemble, swiftly facilitating capillary absorption. In contrast, insulin glulisine is formulated without zinc ions and incorporates substitutions that enhance stability, eliminating the need for a hexameric assembly [70,81,82]. Ongoing crystallographic studies of glulisine aim to refine its formulation further.

Advancements in “smart” insulin pumps require ultrarapid analog formulations, which are crucial for robust continuous glucose monitoring (CGM)-based pump control algorithms. Various strategies have been explored, including heating pads, enzyme hyaluronidase co-injection, needle-free jet injection, and microneedle patches [76,83]. Inhalable powdered insulin monomers with rapid onset and short duration have received regulatory approval. Further formulation enhancements, such as active excipients and synthetic copolymers, seek to improve hexamer disassembly rates and boost local blood flow. The investigated ultrafast, ultraconcentrated insulin analog formulations aim to extend pump therapy to individuals with significant insulin resistance.

## 4. Photoactivatable Drugs

**Figure 4:**
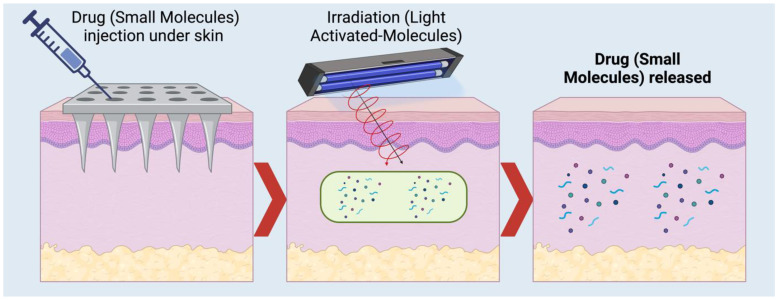
Basic concept of a photoactivatable drug scheme. Injection of modified small molecules/drugs into the body and continued activation using light at a certain wave to release the active molecules/drugs. Created using Biorender Premium, Licensed by Fahrul Nurkolis.

The recent revival in organic photochemistry has been driven by the recognition that numerous visible light-absorbing chromophores can efficiently convert photonic energy into chemically sound potential [41,45,84], as shown in Figure 4. Photocatalysis, a vital aspect of this revival, provides a convenient method for generating diverse open-shell intermediates, and research over the past decade has focused on developing new photoredox transformations based on their reactivity. These reactions involve balanced photocatalyst-mediated electron transfer steps initiated with photo-induced one-electron oxidation or reduction. Successful applications of photoredox catalysis were initially net redox-neutral, primarily due to the requirement for complementary redox steps to regenerate the photocatalyst [85,86].

Recent efforts have developed net oxidation and reduction reactions, harnessing unique reactivity accessible via photoredox catalysis [87,88,89,90]. Developing such transformations has been challenging, as photocatalyst regeneration necessitates stoichiometric terminal oxidants or reductants. Identifying compatible redox reagents is crucial, considering the photocatalyst, organic substrates, photogenerated intermediates, and the final products [91,92,93,94]. Tertiary amines and dihydropyridines have been recognized as practical and inexpensive terminal reductants, facilitating the early development of various photocatalytic reduction reactions [95,96,97].

One notable example of a photoactivatable drug is “Visudyne” (verteporfin). Visudyne is used in photodynamic therapy (PDT) for the treatment of certain eye conditions, particularly age-related macular degeneration (AMD) [98,99,100]. In this context, Visudyne is injected into the bloodstream and accumulates in abnormal blood vessels in the eye. When exposed to light of a specific wavelength, Visudyne becomes activated, producing a chemical reaction that forms reactive oxygen species (ROS). These species then damage and selectively destroy the abnormal blood vessels, helping to halt the progression of AMD.

Another example of a photoactivatable drug is “Amphinex” (also known as hexaminolevulinate or HAL), which is used in photodynamic therapy (PDT) for certain types of cancer, such as bladder cancer [101,102]. Amphinex is administered to the patient and preferentially accumulates in cancer cells [103,104]. Upon exposure to light, specifically blue light, Amphinex undergoes photoactivation. This activates the drug, leading to the generation of ROS. These ROS induce cell death, selectively destroying the cancer cells [105,106,107,108]. This targeted approach minimizes collateral damage and enhances the precision of the therapeutic intervention. One challenge in cancer treatment is achieving sufficient light penetration into tissues for effective drug activation. Tumor heterogeneity and the development of resistance mechanisms can also reduce the overall effectiveness of photoactivatable drugs [109,110,111].

## 5. Light-Activated Insulin and Its Action in Blood Glucose Regulation

**Figure 5:**
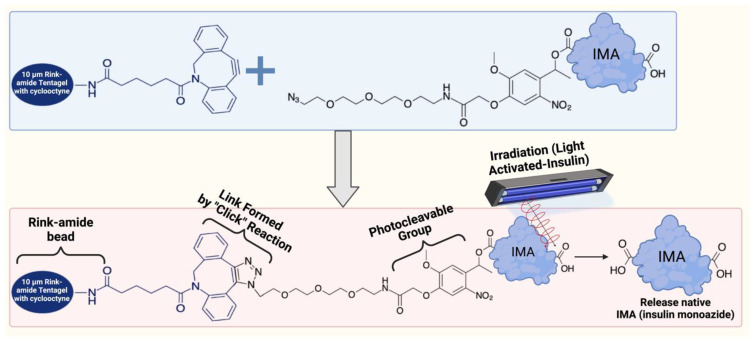
Conceptual biosynthesis framework of light-activated insulin. This figure was modified from Sarode et al. (2016) [115]. The rink amide TentaGel (10 μm) resin added to a cyclooctyne strain was used in the biosynthesis of photoactivated depot material, which was then reacted with insulin monoazide (IMA) containing one photocleavable group. The final material was photolyzed to release native insulin and created with Biorender Premium, licensed by Fahrul Nurkolis.

The novel idea of light-activated insulin is revolutionary for controlling blood glucose levels, providing a fresh approach to treating diabetes [11,112]. This approach entails producing small-molecule biosimilars or insulin derivatives that are both safe and exhibit reduced side effects [112]. These compounds may be triggered by light exposure [11,112]. Interdisciplinary cooperation and improved scientific methodologies are necessary to develop insulin derivatives. A potentially practical approach, shown in Figure 5, involves chemically bonding photoremovable-protecting groups (PPGs) to insulin. This method efficiently encapsulates the insulin, preventing its activity until it is subjected to light, triggering activation of its function [113]. Ideally, this enclosed insulin should exhibit great photosensitivity to non-harmful light wavelengths, enabling prompt and accurate insulin release upon light exposure [113]. It should also avoid unintended consequences and maintain complete stability under physiological settings without light [113]. Furthermore, the sensitivity of insulin release using two-photon excitation (2PE) is essential for precisely localizing the release at the subcellular level [114].

**Figure 6:**
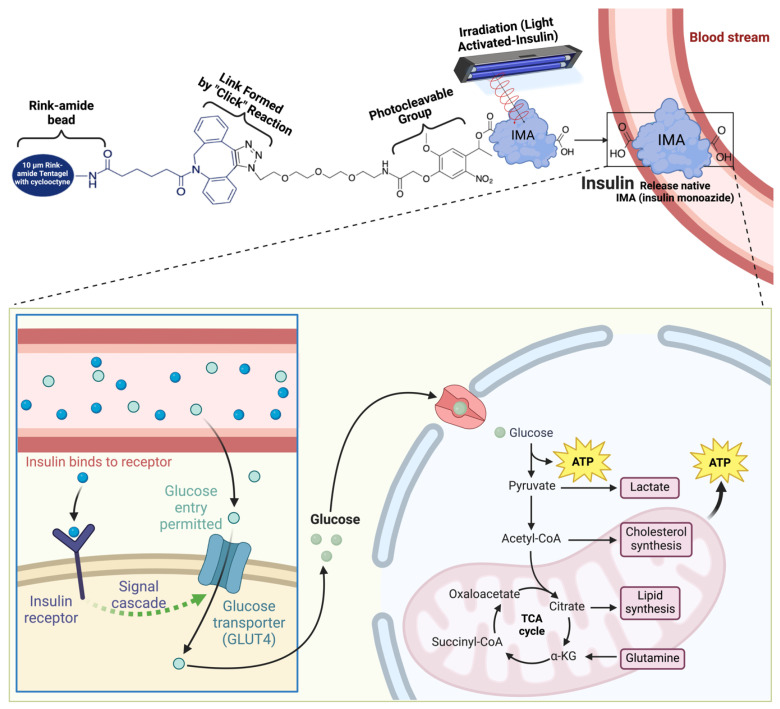
Possible biomechanism of light-activated insulin. TentaGel’s rink amide resin added to the cyclooctyne strain was used in the biosynthesis of photoactivated depot material, which was then reacted with insulin monoazide (IMA) containing one photoactivated group. The last ingredient was photolyzed using certain waves to release native insulin or the active version of insulin to perform its role in blood sugar regulation. Created using Biorender Premium, licensed by Fahrul Nurkolis.

When these modified versions of insulin are created, known as small-molecule derivatives or biosimilars, it is essential to investigate the dynamics of insulin ligand-gated ion channels and the release rate constants, even if they have a poor photolysis quantum yield and molar absorptivity. These parameters are crucial for investigating light-induced insulin. The primary focus is on these molecules’ applicability for controlling insulin release at a specific location and time. This control may be achieved by both single-photon (1PE) and two-photon (2PE) excitation in biological systems [114], with a particular emphasis on regulating blood glucose levels. Figure 6 presents a comprehensive depiction of these processes.

Furthermore, research that highlights the effectiveness of light-triggered insulin, including its duration, wavelength, and procedures, may involve experimental methodologies involving other hormones besides insulin, such as serotonin. The insights gained from these models may be beneficial when evaluating ideas about insulin. After determining the kinetics and release rate constants of insulin ligand-gated ion channels by studying probable small-molecule derivatives or biosimilars of insulin, the subsequent task is to determine the activation wavelength. As the conceptual pictures show, this stage is crucial for achieving an optimal decrease in blood glucose.

This paper delves into a crucial question: “What is the rationale behind developing small-molecule derivatives or biosimilar insulin when endogenous insulin already exists in the body?” Why not subject it directly to light exposure? The solution rests in creating altered insulin molecules that could be triggered by specific light frequencies, reducing adverse effects, and perhaps focusing on RNA and epigenetic elements.

The novel light-activated insulin method has notable advantages over conventional insulin treatments. It offers unparalleled accuracy in controlling blood glucose levels, creating new avenues for diabetes care, and possibly increasing the range of treatment choices for individuals with diabetes.

## 6. Challenges, Future Directions, and Implications of Light-Activated Insulin in the Future

As we explore the possibilities of light-activated insulin, it becomes clear that thorough experimental studies and development are crucial. The importance of these cannot be overstated. The primary focus should be identifying small-molecule insulin analogs or derivatives that light can stimulate. Essential areas to investigate include the precise wavelengths necessary for activating caged insulin, techniques for delivering caged insulin to the body that are both effective and minimally invasive, and approaches for transitioning light-activated insulin from small-scale laboratory use to worldwide commercial availability, progressing from molecular findings to consensus among epidemiologists. Moreover, it is crucial to acknowledge and develop ethical protocols for using photoactivatable medications to guarantee patient well-being and safety.

Emphasizing rules and safeguards is an essential aspect of this area of research. To guarantee the safe use of this revolutionary technology, we must properly address any potential concerns regarding side effects. The innovation described here necessitates ongoing experimental studies by multidisciplinary teams of experts in biology, chemistry, physics, pharmacy, and medicine. Collaboration is crucial for making advancements in developing and implementing light-activated insulin. This reflects researchers’ duties towards humanity and their commitment to Sustainable Development Goal 3 [116].

Furthermore, it is essential to address the financial ramifications of using this technology. Although the advantages of light-activated insulin are clear, its affordability remains a major issue. It is imperative to devise strategies to guarantee the availability of this potentially transformative therapy to patients in need, regardless of their financial circumstances. Ensuring broad accessibility will be crucial for effectively implementing light-activated insulin as a regular treatment method in diabetes care.

## 7. Advantages of Light-Activated Insulin

The introduction of light-activated insulin changes how diabetes is treated, providing several notable benefits compared to conventional methods of administering insulin via injections or oral medications. In a situation where light-activated insulin is easily accessible, its advantages are clear, as shown in Table 1.

One key benefit of light-activated insulin is that it is minimally invasive. Light-activated insulin can be provided with minimum physical intrusion, improving patient comfort and compliance. This is in contrast to injections, which sometimes cause pain, and oral drugs, which may have gastrointestinal adverse effects [117,118,119]. This approach dramatically alleviates the physical and psychological strain on people who need regular insulin injections.

Another significant advantage is the decreased likelihood of adverse consequences. Conventional insulin treatments may sometimes result in hypoglycemia or other problems [119]. Light-activated insulin is anticipated to mitigate these hazards by providing accurate regulation and localized effects. The reduced adverse effects may be attributed to the method’s natural ability to regulate insulin release precisely, enabling more precise management of blood glucose levels.

Moreover, light-activated insulin can target RNA and epigenetic factors specifically. This has the potential to provide more individualized and efficient ways of treating diabetes, targeting not just the symptoms but also the fundamental biological origins of the disease. Although up until now there have been no studies that report RNA targeting by light-activated insulin, there have been many studies showing that light-activated drugs can hit RNA and epigenetic targets in cancer [84,109,111], which is an opportunity to be explored in experimental studies on T2D for scientific evidence.

The use of covalent modifiers in light-activated insulin also enables the modification of insulin in a manner that permits precise control of its activity via light exposure. This allows for more accurate control of insulin function, guaranteeing the timely release of the appropriate quantity. This can result in a closer replication of the body’s natural insulin response.

Ultimately, the advancement of light-activated insulin has the potential to transform diabetes management completely. The benefits of this treatment compared to standard/conventional insulin therapy, as shown in Table 1, include reduced invasiveness, fewer adverse effects, the ability to target RNA and epigenetic variables, and the use of covalent modifiers for precise regulation. These advantages can lead to a more efficient, user-friendly, and advanced approach to diabetes care.

## 8. Another Possible Mechanism

Within the field of diabetes care, there is ongoing research into the use of light-based therapies. This area has excellent potential for theoretical advancements, going beyond the current method of using biosimilar insulin. Figure 6 outlines the possible biomechanism of light-activated insulin.

One excellent theoretical development is the optogenetic manipulation of pancreatic beta cells [120]. This approach involves genetically modifying pancreatic beta cells to respond to light stimuli, resulting in controlled insulin release. If implemented, this technology has the potential to replicate natural glucose-regulating systems accurately.

Moreover, using nanoparticles to enhance sensitivity to light is an innovative approach to non-invasive glucose control [121]. Theoretical nanoparticles, intended for intravenous delivery and activation using precise light wavelengths, have the potential to stimulate insulin production. This technique would fundamentally change how glycemic levels are managed.

Moreover, developing implantable devices that can be activated by external light sources is essential in this field [122]. These proposed technologies have the potential for uninterrupted and automated adjustment of insulin release or function, suggesting a future marked by improved uniformity and regulation in insulin administration methods.

Theoretical debates on photoresponsive insulin molecule engineering are also ongoing. These hypothetical molecules, which include components that respond to light, are proposed to change their functional state upon light exposure, with a direct and precise control mechanism for insulin.

Equally persuasive is the concept of IRs that are triggered by light. If these theoretical constructions are realized, light-based interaction might adjust or stimulate them, enhancing the body’s response to insulin and improving communication among cells. These developments might be crucial for tackling pathological conditions, such as insulin resistance.

Microfluidic devices controlled by photonic stimuli are also a crucial aspect of this story [123]. These devices, now in the conceptual phase, are designed to provide insulin in direct correlation to specific glycemic indices or photonic signals, thus enabling the development of accurate glucose control methods.

Furthermore, the theoretical methodology of using photochemical processes to facilitate insulin release from carrier molecules is a novel mechanism for precise and focused administration. This approach, using photonic-triggered chemical processes to release insulin at specific anatomical locations, is a more effective and targeted delivery method.

Finally, the possibility of influencing gene expression using light, especially techniques similar to optogenetics [124], is a revolutionary approach to regulating insulin production. This hypothesis suggests that insulinogenic cells may adjust their insulin production in response to the changing physiological needs of the organism.

Now under theoretical investigation, these hypothetical light-based processes provide a range of revolutionary opportunities for diabetes care. Every theoretical framework provides a distinct solution to the fundamental limits of conventional insulin treatments for a more effective, adaptable, and patient-focused approach to managing diabetes. Even though up to now the existing research states that this has very little to no side effects, monitoring the risks posed by photoactivable drugs to the patient’s body should be a concern for the future.

## Figures and Tables

**Table 1:** Light-activated insulin vs. injected/oral insulin.

Scopes	Light-Activated Insulin	Conventional Injected/Oral Insulin
Economic	One injection that is long-lasting (affordable)	Injected frequently (high cost)
Safety	Fewer adverse effects (low pain tolerance)	Pain and gastrointestinal adverse effects
Clinical–physical intrusion	Minimally invasive	Invasive
Target	Covalent modifiers for precise regulation; RNA and epigenetic factors related to diabetes	It only increases insulin levels in the bloodstream

## Data Availability

Data are contained within the article.
